# The Diagnostic Function of OCT in Diabetic Maculopathy

**DOI:** 10.1155/2013/434560

**Published:** 2013-11-28

**Authors:** Bartosz L. Sikorski, Grazyna Malukiewicz, Joanna Stafiej, Hanna Lesiewska-Junk, Dorota Raczynska

**Affiliations:** ^1^Department of Ophthalmology, Nicolaus Copernicus University, ul. M. Sklodowskiej-Curie 9, 85-090 Bydgoszcz, Poland; ^2^Department of Ophthalmology, Medical University of Gdansk, ul. M. Smoluchowskiego 17, 80-214 Gdansk, Poland

## Abstract

Diabetic maculopathy (DM) is one of the major causes of vision impairment in individuals with diabetes. The traditional approach to diagnosis of DM includes fundus ophthalmoscopy and fluorescein angiography. Although very useful clinically, these methods do not contribute much to the evaluation of retinal morphology and its thickness profile. That is why a new technique called optical coherence tomography (OCT) was utilized to perform cross-sectional imaging of the retina. It facilitates measuring the macular thickening, quantification of diabetic macular oedema, and detecting vitreoretinal traction. Thus, OCT may assist in patient selection with DM who can benefit from treatment, identify what treatment is indicated, guide its implementing, and allow precise monitoring of treatment response. It seems to be the technique of choice for the early detection of macular oedema and for the followup of DM.

## 1. Introduction

Diabetic retinopathy is the name given to the changes in the retina, which develop over a period of time in diabetics. It remains one of the major causes of new-onset visual loss in developed countries. If the central part of the retina (i.e., the macula) is involved, it is referred to as diabetic maculopathy. This is the most common cause of vision impairment in individuals with diabetic retinopathy [1]. The traditional approach to diagnosis of diabetic maculopathy includes fundus ophthalmoscopy and fluorescein angiography (FA) [2]. The Early Treatment Diabetic Retinopathy Study (ETDRS) identified stereoscopic slit-lamp biomicroscopy and stereo colour fundus photography as standard methods of macular thickness assessment utilized in order to determine whether the treatment should be commenced as they defined the clinically significant macular oedema (ETDRS report number 10, 1991). However, these methods are subjective and relatively insensitive to small changes in retinal thickness and, therefore, may be unable to identify mild or localized macular thickening [3]. They also do not provide any data on retinal morphology and blood flow. On the other hand, FA is a highly effective test of evaluating retinal blood vessels, macular perfusion, and pattern of leakage causing the oedema. Although very useful clinically, it also does not contribute much to the evaluation of retinal morphology and its thickness profile.

In 1991 the researchers from Massachusetts Institute of Technology and Harvard University patented the technique of optical coherence tomography (OCT), which was a major breakthrough in ophthalmic diagnostics (US5321501 A, Swanson EA, Huang D, Fujimoto JG, Puliafito CA, Lin CP, Schuman JS. Method and apparatus for optical imaging with means for controlling the longitudinal range of the sample). The first paper to present the potential of the new diagnostic method was published in the same year [4]. Four years later the first paper was published which described the use of OCT in diagnosis of macular diseases [5]. Nowadays, OCT is one of the fundamental diagnostic imaging techniques in ophthalmology. It is an essential compliment to ophthalmoscopy and FA in patients with diabetic maculopathy.

The purpose of this paper is to provide an overview of clinical utility of OCT in retinal assessment of diabetic patients.

## 2. OCT Principles and Interpretation

### 2.1. OCT Principles

OCT enables obtaining the high resolution (few micrometres) cross-sectional images (tomograms) of the human retina in a noninvasive manner [6]. Retinal morphology is reconstructed based on the analysis of backscattered or reflected light. In contrast to classic fundus photography taken with fundus camera, OCT also provides information on the depth that the scattered light comes from. If light is reflected by the deeper retinal layers, it has to go a longer way to return to the detector compared to the light reflected from more superficial layers. That is why it takes the light longer to return from deeper layers. This feature makes it possible to precisely determine what retinal depth (i.e., layer) the particular signal comes from. Therefore, OCT resembles ultrasound imaging with the only difference consisting in utilizing light instead of sound. The use of light gives OCT higher axial resolution compared to any other imaging techniques currently used in clinical medicine.

In classic OCT setup the light emitted by the superluminescent diode is directed to the beam splitter which splits it into two equal beams. One of them is projected onto the reference mirror, the other one onto the retina, and is backscattered from its morphological elements. The light waves reflected back from the retina and the reference mirror are superposed. The wave interference may occur only when the optical path between the beam splitter and the mirror is equal to the distance between the splitter and one of the surfaces reflecting the light within the retina. In that case, the detector will record the change in the light intensity. In order to detect other reflecting surfaces, the position of the reference mirror is moved in relation to the beam splitter. The OCT technique described above is referred to as Time Domain OCT (TDOCT) due to the fact that the information on retinal morphology along the scanning beam is obtained by recording the optical signal when the mirror is moved. It was the first OCT technique described in 1991.

An alternative solution is Frequency Domain OCT (FDOCT). It differs from TDOCT in how the sample image is constructed. This technique required the reference arm to be held fixed, and the optical path length difference between sample and reference reflections is encoded by the frequency of the interferometric fringes as a function of the source spectrum. There are two practical implementations of FDOCT. The first is Spectral Domain OCT (SDOCT) in which interferometric signal is detected using the spectrometer equipped with a line of light sensitive elements [7]. The other method is a Swept Source OCT (SSOCT) utilizing swept tunable lasers and a standard photodiode detector [8]. As in FDOCT, the reference mirror remains fixed, the better mechanical stability of the system is achieved. Additionally, the interferometric signal created by mixing the sample and reference light is sampled as a function of wavenumber and yields an entire depth scan at the same time. This makes it possible to achieve several hundred-fold increase in speed and sensitivity of scanning compared to TDOCT. As a result, significant motion artefacts are avoided and multiple measurements can be taken in a short time enabling the three-dimensional retinal scanning. The OCT images can be also acquired at the video-rate and the measured structures observed in real time. The first commercially available SDOCT device (Copernicus, Optopol SA, Poland) was launched in 2006.

### 2.2. Data Visualization

The term *reflectivity* is used in OCT technique as an equivalent of *echogenicity* in ultrasonography. It means the ability of the analysed structure to reflect the light waves. The areas showing reduced reflectivity are referred to as hyporeflective, whereas the increased reflectivity regions are referred to as hyperreflective. The reflectivity in a grey-scale is proportional to tissue brightness observed in OCT. The higher the reflectivity is, the closer to white the colour will be. In order to effectively visualise subtle structures in OCT scans, the false colour scale is often clinically used, in which the individual colours are purely conventional. Usually white and red represent highest intensity signal, whereas black and blue correspond to the lowest intensity signal. However, such approach has a disadvantage, namely, possible occurrence of artefacts [9]. If signal intensity changes, the colour of a given structure on OCT scan may also change.

**Figure 1:**
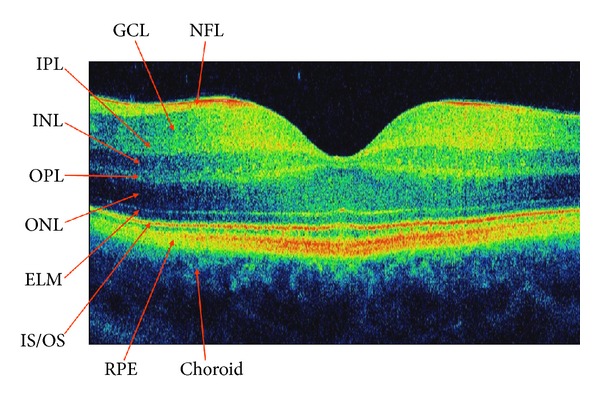
SDOCT cross-sectional image of a normal human macula. NFL: nerve fibre layer, GCL: ganglion cell layer, IPL: inner plexiform layer, INL: inner nuclear layer, OPL: outer plexiform layer, ONL: outer nuclear layer, ELM: external limiting membrane, IS/OS: the junction between the inner and outer photoreceptor segments, RPE: retinal pigment epithelium.

**Figure 2:**
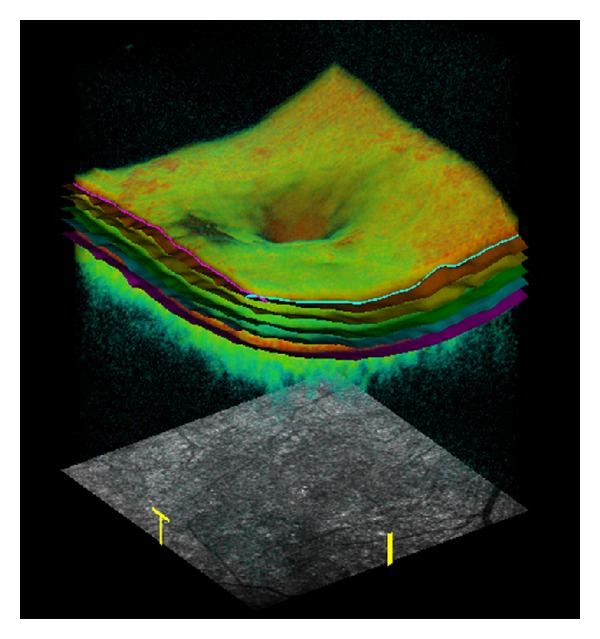
Volume rendering of the macula from the three-dimensional SDOCT data. Segmentation result showing the intraretinal layers.

The OCT results are presented as an axial scan, referred to as an A-scan, similarly like in ultrasonography. It presents retinal reflectivity at different depths along the scanning beam axis. It is acquired by presenting the amplitude of the back-scattered light as a function of echo delay time. As the scanning beam moves along the retina, many A-scans are acquired, which form the tomogram, that is a B-scan (Figure 1). It presents the cross section of the retina in a plane perpendicular to its surface. The set of many consecutive B-scans is assembled into a 3D reconstruction of retinal structure (Figure 2).

**Figure 3:**
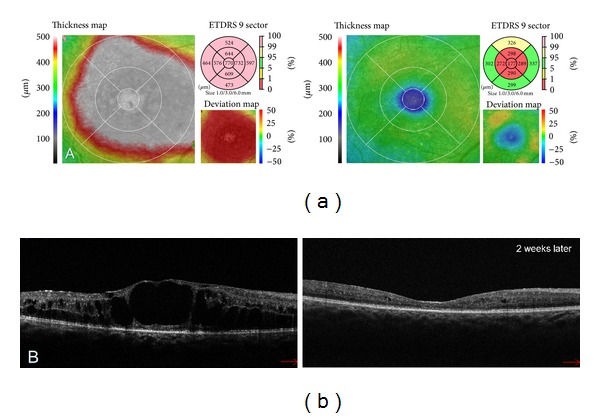
(a) Colour-coded macular thickness maps revealing the change in retinal thickness between the examinations. The second map was obtained 2 weeks after treatment with Ranibizumab. Resolution of DME is evident but the corresponding thickness deviation map shows that the anatomical improvement is also related to the central macular atrophy which may limit the visual acuity. (b) Corresponding SDOCT tomograms presenting the disappearance of nearly all intraretinal cystoid spaces.

The software in-built in commercially available OCT devices makes it possible to carry out a quantitative data analysis. The results of such analyses primarily include total retinal and individual layers thickness maps as well as macular volume maps. Retinal thickness is usually calculated for central fixation point, 9 ETDRS-like macular regions, and total macular thickness (Figure 3). Retinal volume is displayed for 9 ETDRS-like macular areas and total macular volume. The OCT data is automatically segmented in order to generate the above maps (Figure 2). When interpreting these maps, one should bear in mind that the artefacts may occur during segmentation, which will lead to improper retinal thickness measurements [10, 11]. Artefacts may arise as a result of poor image quality, eye movement during measurements, and retinal pathologies interfering with automated segmentation (e.g., retinal pigment epithelial detachment, subretinal fluid, fibrosis, or haemorrhage). OCT maps may be compared to normative data, including age, sex, and race (Figure 3) [12–14]. It should be noted that different OCT devices have different in-built normative databases. Therefore, direct comparisons of maps generated by different OCT devices are pointless [15, 16]. The quantitative monitoring of retinal thickness in a given patient requires using the same OCT device model for all follow-up examinations.

### 2.3. Basic OCT Interpretation of Normal Macular Morphology

The interpretation of OCT image is based on analysing tissue reflectivity [17]. It is quite intuitive, but prior to discussing the aspects of the retinal architecture seen in OCT, the physical foundations of the obtained images should be explained. The OCT reflects the optical properties of the imaged tissue. That is why the OCT images should not be interpreted indiscriminately as histologic specimens. In histopathological examination, the contrast between the individual structures is obtained owing to tissue staining. Different stains show the affinity to different morphological elements, for example, cell nuclei. On the other hand, particular colours on OCT cross-sectional image correspond to different signal intensity levels. However, since retinal OCT images highly resemble histologic specimens, despite the above difference, and OCT is a noncontact examination, it can be seen as an optical biopsy, which does not require tissue excision in order to analyse its structure.

Histologically, the retina consists of 10 layers, 4 of them being cellular and 2 neuronal junctions. The axonal layers, that is, the nerve fibre layer and the plexiform layers, are capable of potent light scatter and appear yellow to red on false-colour OCT images. The light scattering potential of nuclear layers is lower, so they are represented as blue and black areas. The first layer visible on OCT images is the internal limiting membrane which appears as a hyperreflective line at the vitreoretinal interface. It is visualized in OCT owing to the increased light scatter between the transparent vitreous and retinal surface. Below, there lies the retinal nerve fibre layer, typically thicker in the nasal macula and capable of potent light scatter. The next imaged structure is a hyporeflective ganglion cell layer. Subsequently, hyperreflective plexiform layers are imaged as well as the inner nuclear layer situated between them, which has a lower light scattering potential. Then, the relatively thick hyporeflective outer nuclear layer is visible with a thin hyperreflective line underneath. This line corresponds to the location of the external limiting membrane (ELM). A distinct bright stria that stretched in front of the RPE demarcates the junction between the inner and outer photoreceptor segments (IS/OS). Due to the increased length of outer cone segments in central fovea, this line is slightly elevated in foveal region. The last of the imaged layers is retinal pigment epithelium (RPE). It contains melanin and is capable of very potent light scattering. On the other hand, Bruch's membrane is too thin to be imaged as a separate structure. Below, choriocapilaries and the rest of the choroid are visible. In the centre of the macula there is thinning of the retina with the absence of the inner layers. It is easily recognized on cross-sectional images by its characteristic depression.

### 2.4. Basic OCT Interpretation of Macular Pathology

Reduced reflectivity is most often cause by intraretinal and subretinal fluid accumulation (oedema, retinal detachment, serous RPE detachment). Pathological features that can be hyperreflective are: hard exudates, calcification, epiretinal and thick vitreous membranes, fibrosis, haemorrhages, RPE hyperplasia, neovascular membranes, atrophy of the retina and RPE causing increased reflectivity of underlying choroid [18].

## 3. OCT Findings in Diabetic Maculopathy

**Figure 4:**
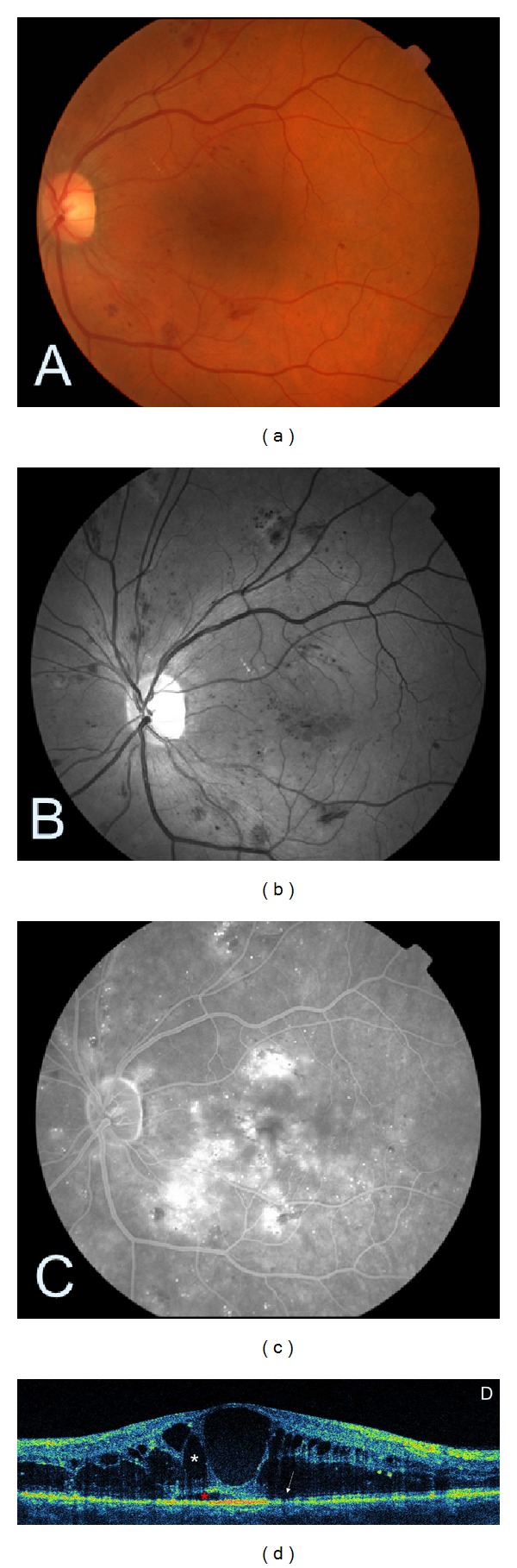
Cystoid DME. (a) Colour fundus photography showing a few intraretinal haemorrhages. (b) The corresponding red-free image of the fundus. (c) Late-stage fluorescein angiogram depicting a cystoid pattern of foveal hyperfluorescence with surrounding diffuse leakage. (d) SDOCT demonstrating hyporeflective cystic spaces within the retina (white asterisk) and subretinal fluid accumulation (red asterisk) consistent with macular oedema. The abnormal reflectivity of photoreceptor layer is indicated by a white arrow.

**Figure 5:**
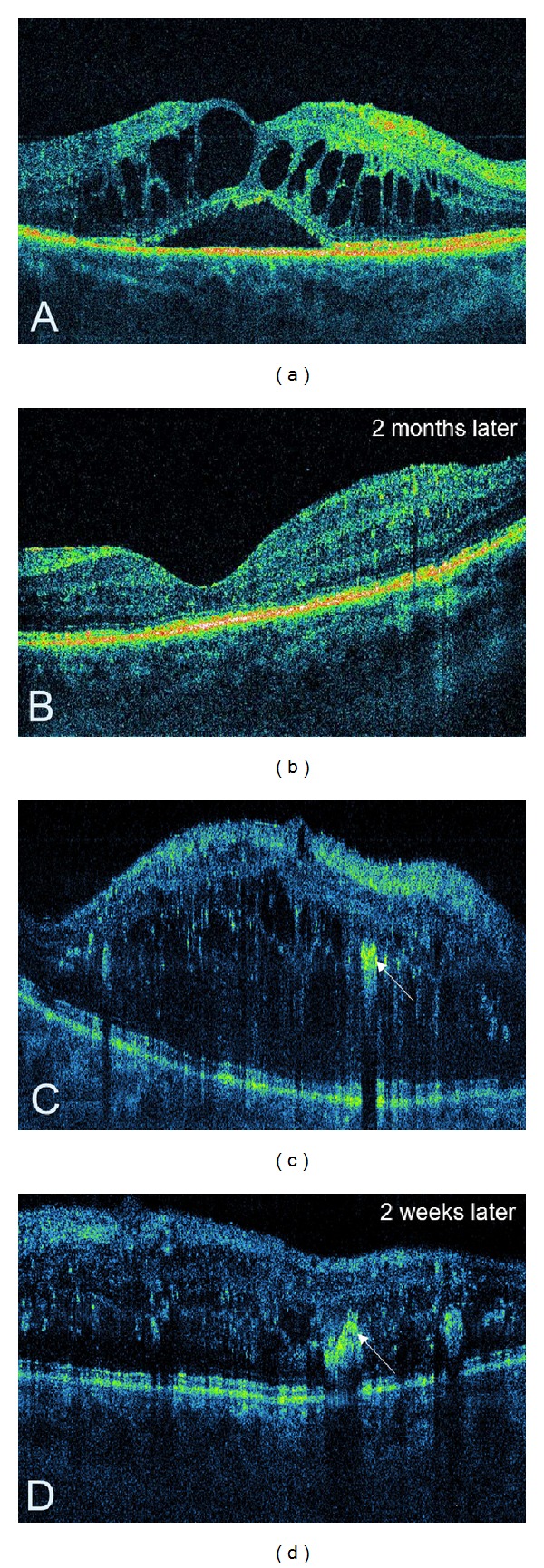
(a, b) Cystoid DME treated with intravitreal steroid injection. (c, d) Diffuse DME treated with Ranibizumab and laser therapy. The arrows point to hard exudates.

OCT enables precise measurement of macular thickness. Thus, it facilitates detecting macular oedema which is the main pathologic feature of diabetic maculopathy. This is defined as any detectable retinal thickening due to fluid accumulation (ETDRS report number 10, 1991). The oedema may be symmetrical or involve only a sector of the macular area. It usually starts as a focal lesion and progresses towards a more diffuse form. In some cases, the macular edges may be thickened, even though the contour of the foveal centre remains normal. Persistent retinal oedema resulting in Muller cell necrosis leads to the formation of cystoid cavities, located mainly in the outer retina (Henle's fibre and outer plexiform layer), and sometimes also in the inner plexiform layer. In the most advanced stages in eyes with well-established long term macular oedema, several central cysts can merge together forming large hyporeflective cavity which contributes to the significant thickening of the fovea (Figure 4). Therefore, the main characteristics of macular oedema in OCT, apart from increased retinal thickness, include intraretinal spaces of reduced reflectivity, disintegration of the layered retinal structure, and usually also flattening of the central foveal depression. In some cases fluid can be seen under the neurosensory retina (Figures 4 and 5(a)) [19]. OCT tomograms can also reveal hard exudates and haemorrhages. They present as small hyperreflective deposits with posterior shadowing (Figures 5(c) and 5(d)).

Intraretinal cysts can differ in size. That is why Koleva-Gorgieva proposed the classification of cystoid diabetic macular oedema (DME) into mild, moderate, and severe according to the size of cystoid spaces [20]. The mild cystoid DME presents with small cysts predominantly in the outer retinal layers. The cystoid spaces in eyes with intermediate and severe cystoid DME are mainly located in the outer layers, predominantly in the fovea. In some cases small cysts in the inner layers can also be found. If the cysts continue to increase, they may occupy the full thickness of the retina, leading to its atrophy and the profound vision loss.

In the past few years, since the introduction of FDOCT, it has become possible to accurately visualize the outer retinal layers. The integrity of these layers has been shown to correlate with retinal function. Several authors have reported that the integrity of ELM and IS/OS junction has a positive correlation with visual acuity [21, 22]. Shin et al. showed that the photoreceptor layer status is closely associated with final visual acuity in DME and that photoreceptor integrity prior to treatment can be predictive of potential visual recovery in DME [23]. Yohannan et al. demonstrated that disruption of IS/OS junction correlates well with a significant decrease in point sensitivity in eyes with DME [24]. Therefore, the assessment of outer retinal layer structure should be a part of a routine evaluation when performing OCT in patients with DME.

**Figure 6:**
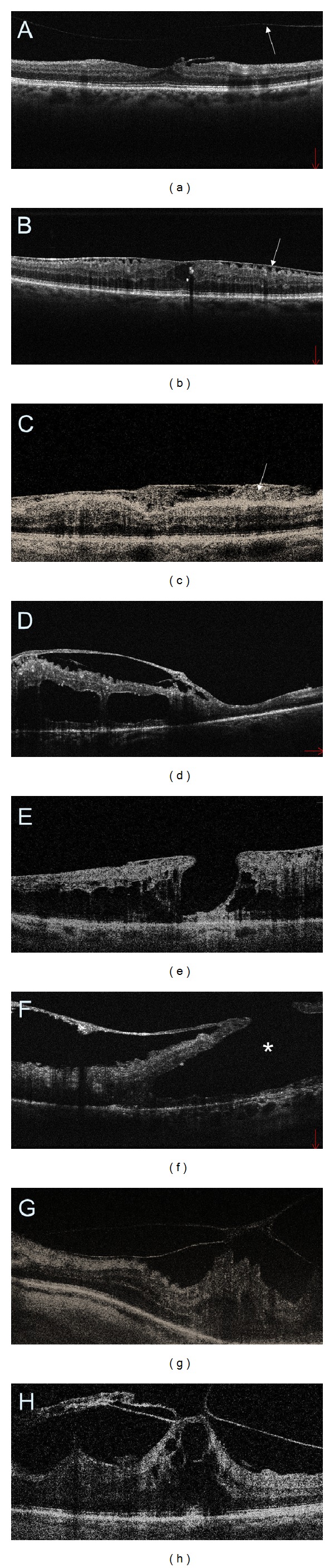
SDOCT cross-sectional images of the macular vitreoretinal interface abnormalities in diabetic retinopathy. (a) Posterior vitreous detachment (arrow) and a small, localized epiretinal membrane (ERM). (b) A thin ERM that is separated from the retinal surface in multiple areas causing distortion to the inner retinal layers and flattening of the central foveal depression. (c) ERM with an extensive fibrosis (arrow). (d) A thick and taut ERM inducing cystoid DME. (e) A thin ERM together with macular pseudohole. (f) Preretinal fibrosis causing the formation of lamellar macular hole (asterisk). (g) Vitreomacular traction. (h) An extensive macular traction with cystoid DME.

**Figure 7:**
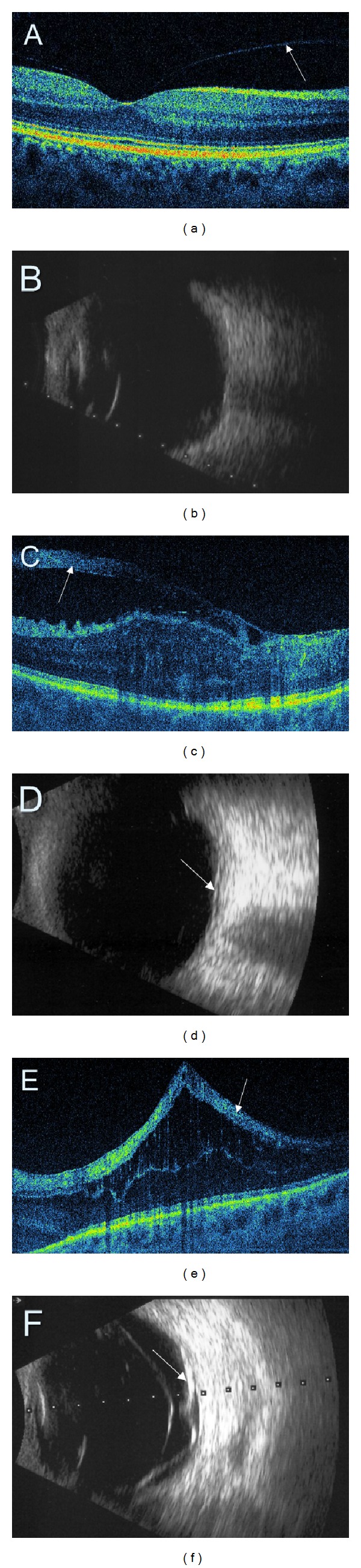
Comparison of retinal SDOCT (a, c, e) with ultrasonography (b, d, f). (a) Perifoveal posterior vitreous detachment (arrow) (PVD). (b) No PVD is detectable on ultrasonography. (c, d) A thick preretinal fibrosis (arrows). (e, f) Tractional macular detachment (arrows).

**Figure 8:**
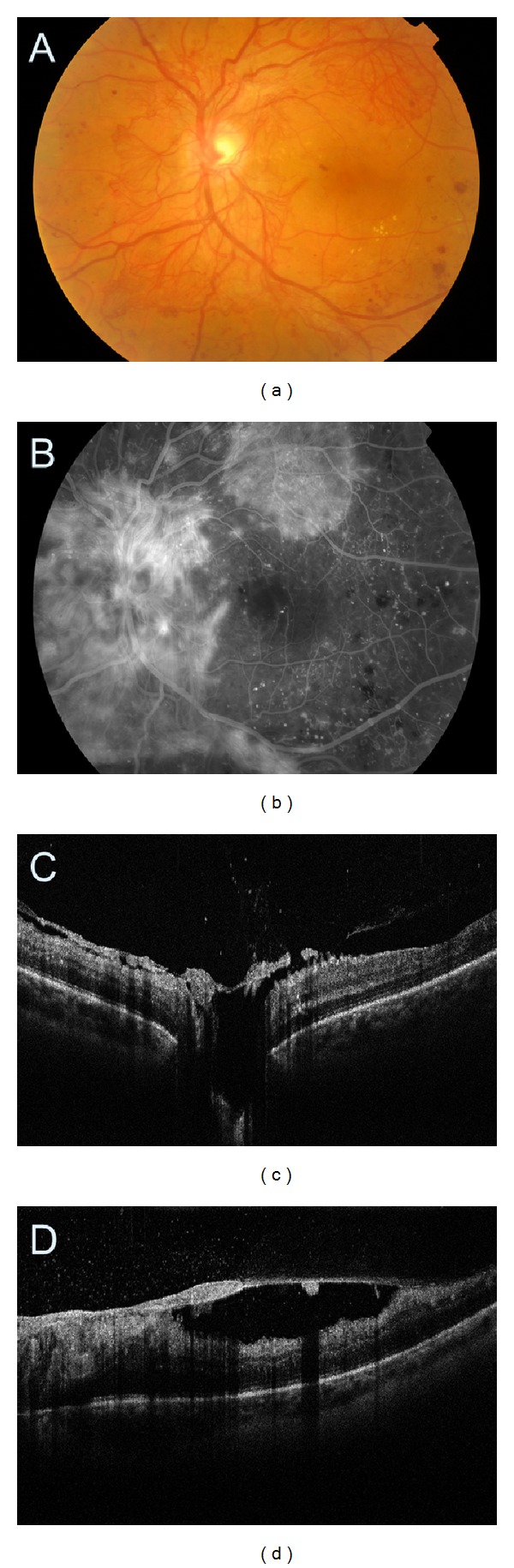
Proliferative diabetic retinopathy with neovascularization of the disc (NVD) and elsewhere (NVE). (a) Colour fundus photography. (b) Fluorescein angiography presenting an extensive leakage at the site of NVD and NVE. (c, d) SDOCT showing preretinal neovascular membrane at the disk and in the superior macula.

**Figure 9:**
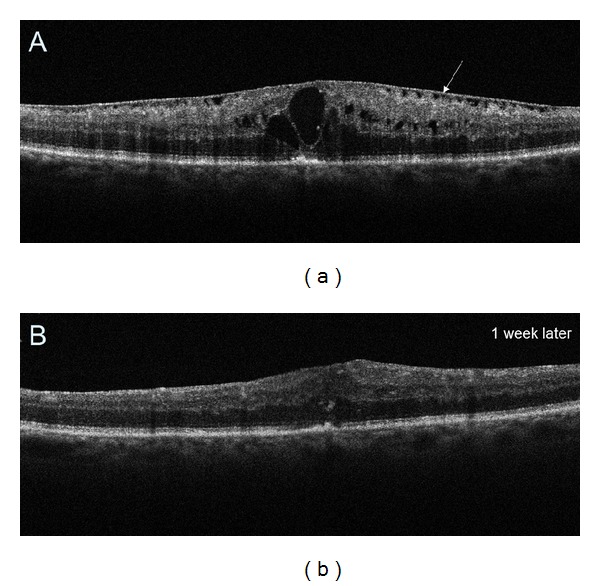
(a) Preoperative SDOCT tomogram demonstrating epiretinal membrane (arrow) and cystoid DME. (b) Postoperative SDOCT image obtained 1 week after vitrectomy showing decreased retinal thickness and no intraretinal cystic spaces. Normal foveal depression is still absent.

**Figure 10:**
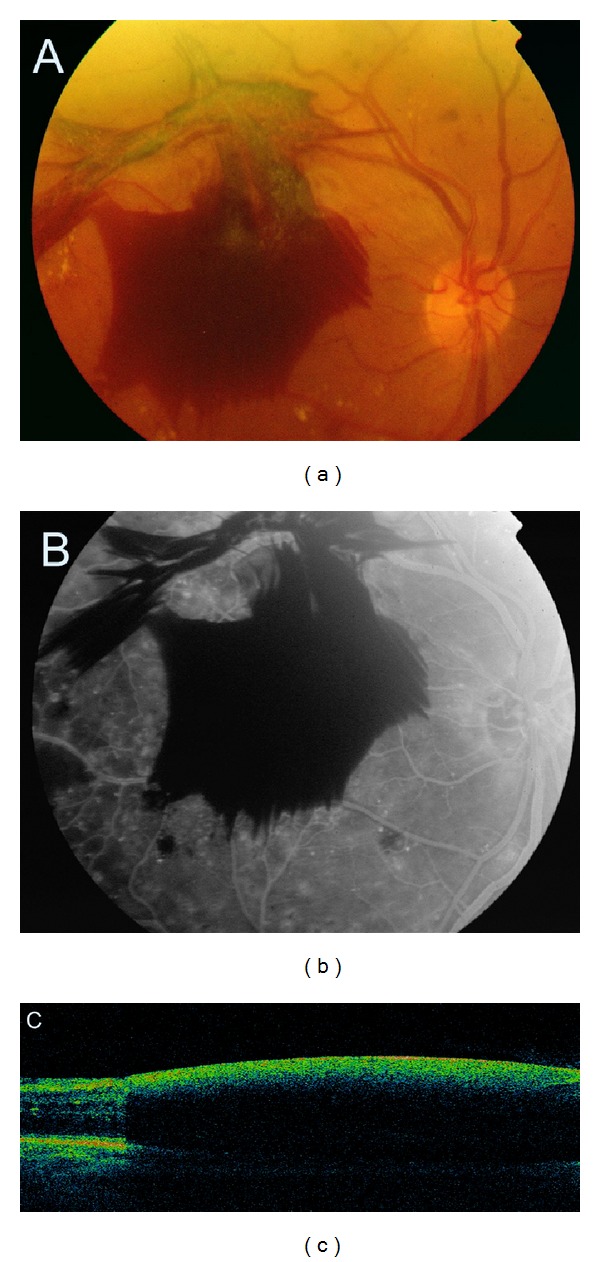
Proliferative diabetic retinopathy with dense preretinal haemorrhage. (a, b) Colour fundus photography and fluorescein angiogram. The view of the retina is obscured by the haemorrhage. (c) SDOCT cross-sectional image of the macula. The optical signal of the retina is shadowed by the haemorrhage.

**Figure 11:**
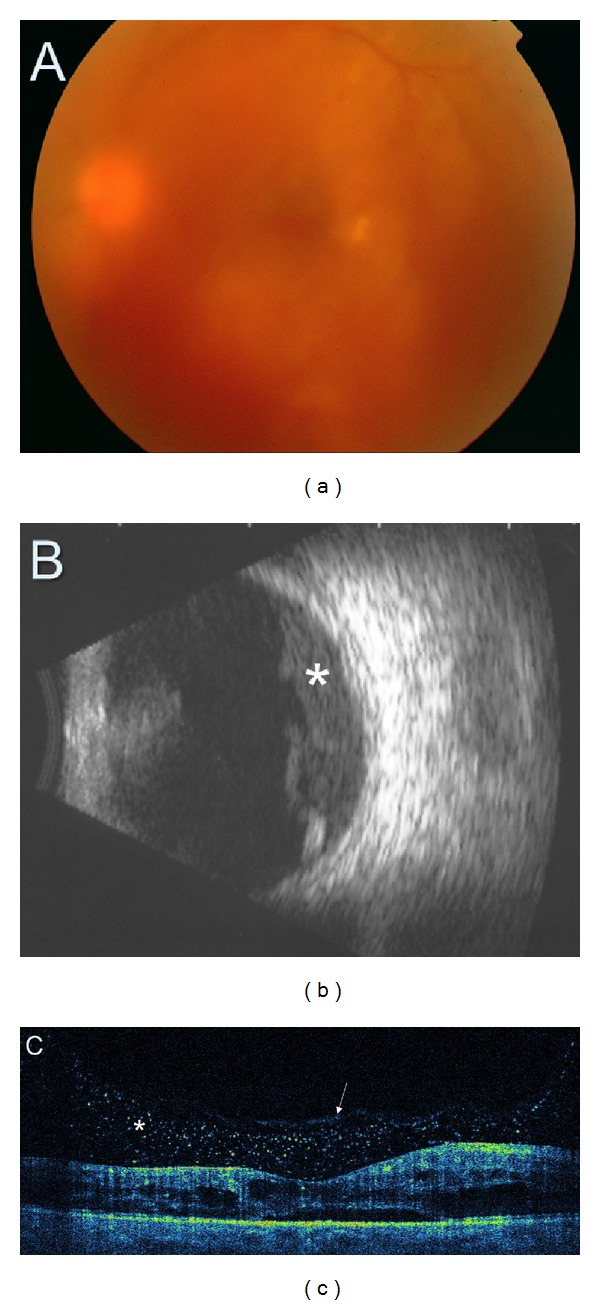
(a) Colour fundus photography demonstrating moderate vitreous haemorrhage obscuring the view of the macula and the posterior pole. (b) Ultrasonography presents preretinal vitreous haemorrhage (asterisk). (c) SDOCT tomogram. Although fundus assessment in ophthalmoscopy is impossible, SDOCT clearly shows cystic DME with preretinal blood accumulation (asterisk). Posterior vitreous face is marked with a white arrow.

**Figure 12:**
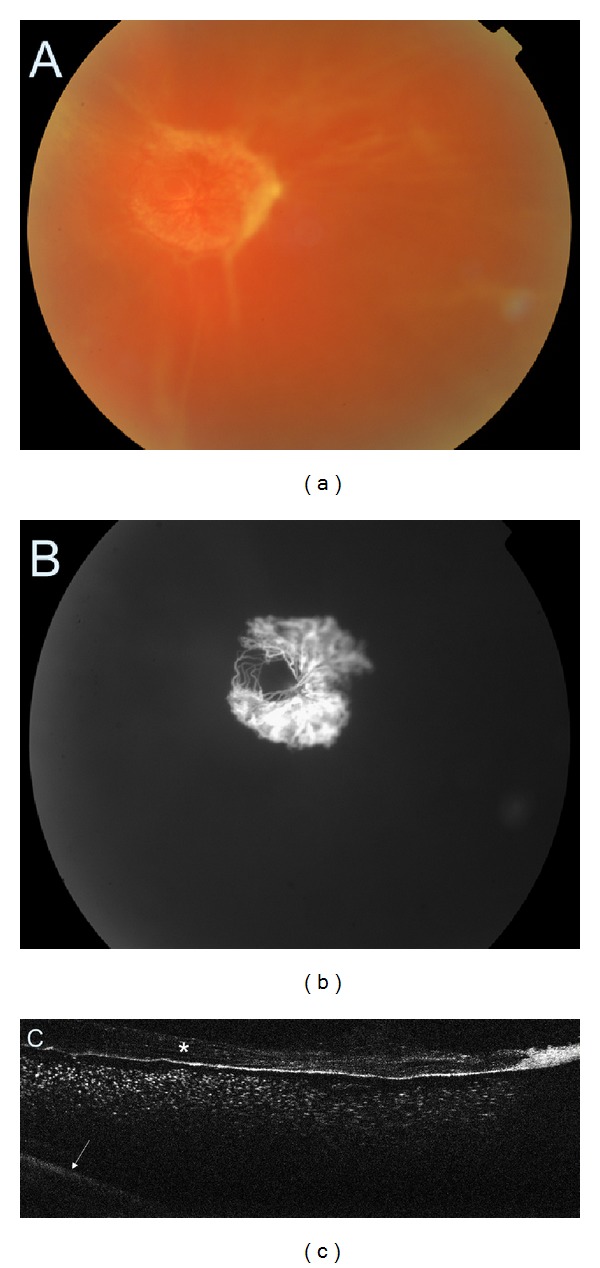
(a) Colour fundus photography of the fibrovascular proliferation. (b) Fluorescein angiography demonstrating the neovascular component of the membrane. (c) SDOCT showing thickened posterior vitreous face (asterisk) with preretinal haemorrhage. The retina is barely visible (arrow).

The ability to visualize the vitreoretinal interface (Figures 6, 7, and 8) is a unique feature of OCT. It allows for macular traction imaging, which may play a role in DME development [25, 26]. The traction may be induced by vitreoretinal interface abnormalities such as incomplete posterior vitreous detachment (PVD) or epiretinal membrane (ERM). If the posterior hyaloid is thin and only slightly detached from the surface of the macula, it is not visible in ophthalmoscopy, but can be easily detected by OCT. The same is true for ERM; if it is thin and does not cause a significant retinal distortion, it can be only visualized using OCT. The detection of clinically significant macular traction may affect therapeutic management of DME. Releasing the traction during vitrectomy may be the best treatment option in those patients [27]. That is why the assessment of vitreoretinal interface is an essential step in macular evaluation in patients with diabetic retinopathy. Moreover, OCT does not only work well as a diagnostic tool in macular traction but may also be used in order to monitor the postoperative morphological outcomes (Figure 9). It can also help identify the postoperative complications of vitrectomy, such as retinal detachment, ERM, and lamellar macular hole formation. The detached posterior vitreous face presents OCT scans as a thin hyper-to-medium reflective horizontal or oblique line in the non-reflective vitreous cavity, above or inserting into the retina. In case of incomplete PVD it may adhere to the foveal or the peripapillary region [28]. ERM on OCT scans presents as a hyperreflective line lying on retinal surface. It can lead to increase in macular thickness, loss of foveal depression, and formation of intraretinal cystoid spaces or pseudoholes (Figure 6). The distinction between ERMs and a PVD is usually made on the basis of reflectivity. The latter typically has a lower reflectivity and less consistent appearance than preretinal fibrosis. OCT can also be used to document the opacity and thickness of ERM, its distance from the surface of the retina, and such effect on the underlying retina as distortion, oedema, or neurosensory detachment. It should be noted that OCT is complementary to ultrasound scanning in evaluation of vitreoretinal interface. Ultrasound scans provide a more complete image of vitreous pathology but at the cost of lower resolution. FDOCT, on the other hand, provides a more detailed image of vitreoretinal interface but limited to a relatively small area (Figure 7). As OCT uses light to acquire the images, if the optic media are opacified and fundus cannot be visualized, the retinal cross sections will not be obtained. The limitations are similar to the ones associated with ophthalmoscopy and FA (Figure 10). However, sometimes OCT images can be acquired in cases where retinal assessment in ophthalmoscopy is impossible (Figure 11). In some cases OCT can even enable assessment of the space located posteriorly to the thin fibrovascular membrane in proliferative retinopathy (Figure 12).

Another particular value of OCT is the possibility of reliable and reproducible retinal thickness measurements (Figure 5). Using the retinal thickness maps, it is possible to monitor DME progression and assess treatment outcomes after laser photocoagulation, intravitreal injections of anti-VEGF and steroids or, as mentioned before, vitrectomy. The obtained results can be compared with the normative database. Owing to retinal thickness maps not only oedema but also atrophy can be detected, which contributes to lack of improvement or even decreased vision after the oedema is resolved (Figure 3). The treatment efficacy in DME should, then, be evaluated in terms of two outcomes: the functional one based on visual acuity measurements and anatomical one assessed in OCT.

## 4. OCT Classification of DME

The first OCT classification of DME presented by Otani et al. was based on retinal morphological changes: sponge-like swelling, cystoid oedema, and serous retinal detachment [29]. Along with the improving OCT technology, subsequent authors proposed more and more complex DME classification systems [30–33]. The classification proposed by Koleva-Georgieva appears to be particularly interesting [34]. It is based on authors' own experience and previously published data. It takes into account several quantitative and qualitative OCT data: retinal thickness, retinal morphology, retinal topography, macular traction, and foveal photoreceptor status.

### 4.1. Retinal Thickness

This includes the following:

1. no macular oedema—normal macular morphology and thickness not reaching the criteria for subclinical DME;
2. early subclinical macular oedema—no clinically detected retinal thickening on ophthalmoscopy, OCT measured retinal thickness exceeding normal +2SDs for central fixation point and fovea;
3. established macular oedema—retinal thickening and evident morphological characteristics of oedema.

### 4.2. Retinal Morphology

This includes the following:

1. simple noncystoid macular oedema—increased retinal thickness, reduced intraretinal reflectivity, irregularity of the layered structure, and flattening of the foveal depression, without presence of cystoid spaces;
2. cystoid macular oedema—the above criteria, associated with presence of well-defined intraretinal cystoid spaces:
  (a) mild cystoid macular oedema—cystoid spaces with horizontal diameter <300 *μ*m,
  (b) intermediate cystoid macular oedema—cystoid spaces with horizontal diameter ≥300 *μ*m <600 *μ*m,
  (c) severe cystoid macular oedema—cystoid spaces with horizontal diameter ≥600 *μ*m, or large confluent cavities with retinoschisis appearance;
3. serous macular detachment—any of the above, associated with serous macular detachment (hyporeflective area under the detached neurosensory retina and over the hyperreflective line of the RPE).

### 4.3. Retinal Topography

This includes the following:

1. nonsignificant macular oedema;
2. clinically significant macular oedema, as defined by ETDRS and evaluated on the OCT retinal topography map.

### 4.4. Presence and Severity of Macular Traction (Incomplete PVD and/or ERM)

This includes the following:

1. no macular traction—presence of complete PVD (Weiss ring detected on ophthalmoscopy), or no PVD (no visible posterior hyaloid line on FDOCT), and no ERM;
2. questionable macular traction—incomplete PVD with perifoveal or peripapillary adhesion and/or globally adherent ERM without detectable distortion of retinal surface contour at the points of adhesion;
3. definite macular traction—incomplete PVD with perifoveal adhesion and/or focal ERM with detectable distortion of retinal contour at the points of adhesion.

### 4.5. Retinal Outer Layers Integrity (IS/OS and ELM)

This includes the following:

1. IS/OS and ELM intact;
2. IS/OS and ELM with disrupted integrity.

## 5. Technological Advances in OCT

Apart from the acquisition of morphological images, OCT can also detect a Doppler frequency shift of reflected light, which provides information on flow and movement [35–37]. Wang et al. reported that reproducible and repeatable measurements of total blood flow can be obtained using Doppler OCT [38]. In another study, Wang et al. compared blood flow in a patient with diabetes and no retinopathy with another patient with treated proliferative retinopathy [39]. The first subject showed a total blood flow value at the lower level of the normal range, whereas the same value in a patient with diabetic retinopathy was lower compared to healthy population. These results clearly indicate that Doppler OCT may play a role in noninvasive assessment of retinal blood flow in diabetic patients.

The OCT can also be used for visualizing the tiny blood vessels within the macula. During the American Academy of Ophthalmology Annual Meeting 2012 we presented a novel method (OCT angiography) for the noninvasive visualization of three-dimensional retinal microcapillary network using intensity-based OCT and also validated its clinical usefulness in retinal vascular diseases including diabetic retinopathy (Sikorski BL, Szkulmowski M, Malukiewicz G, Kowalczyk A, Wojtkowski M. Noninvasive visualization of 3D retinal microcapillary network using OCT. PO 263, AAO 2012, Chicago). OCT angiography proved to be capable of showing 10 micron blood vessels, revealing the vascular nonperfusion and identifying microexudates which were not otherwise visible on clinical examination and fundus photography. It also highly correlates with FA. Moreover, OCT angiography can show even more capillaries in the pericentral macula than FA and allows discerning and visualizing separately the superficial and deep capillary plexus. Therefore, we believe that classic structural OCT examination together with OCT angiography may provide a comprehensive solution in a single imaging modality in patients with diabetic maculopathy.

## 6. Conclusion

OCT can perform micrometre-resolution, cross-sectional imaging of the retina that closely approximates its histological layers. One of the huge advantages of OCT is that patients find this procedure very comfortable because it is noncontact and the measurement time is very short. In patients with diabetic retinopathy OCT can be successfully utilized as an objective monitoring technique of the macular thickening before and after therapy. Thus, it facilitates quantification of retinal oedema. OCT is also very useful for vitreous assessment, showing whether it is attached or detached from the macula. It is helpful in detecting vitreoretinal traction that may not have been identified clinically.

To summarize, OCT may assist in patient selection with diabetic maculopathy who can benefit from treatment, identify what treatment is indicated, guide its implementing, and allow precise monitoring of treatment response. OCT also helps to understand the anatomy of DME and the intraretinal damage. It seems to be the technique of choice for the early detection of macular oedema and for the followup of diabetic maculopathy.

## References

[1] Williams R, Airey M, Baxter H, Forrester J, Kennedy-Martin T, Girach A (2004). Epidemiology of diabetic retinopathy and macular oedema: a systematic review. *Eye*.

[2] Kiri A, Dyer DS, Bressler NM, Bressler SB, Schachat AP (1996). Detection of diabetic macular edema: nidek 3Dx stereophotography compared with fundus biomicroscopy. *American Journal of Ophthalmology*.

[3] Hee MR, Puliafito CA, Wong C (1995). Quantitative assessment of macular edema with optical coherence tomography. *Archives of Ophthalmology*.

[4] Huang D, Swanson EA, Lin CP (1991). Optical coherence tomography. *Science*.

[5] Puliafito CA, Hee MR, Lin CP (1995). Imaging of macular diseases with optical coherence tomography. *Ophthalmology*.

[6] Wojtkowski M, Srinivasan V, Fujimoto JG (2005). Three-dimensional retinal imaging with high-speed ultrahigh-resolution optical coherence tomography. *Ophthalmology*.

[7] Wojtkowski M, Leitgeb R, Kowalczyk A, Bajraszewski T, Fercher AF (2002). In vivo human retinal imaging by Fourier domain optical coherence tomography. *Journal of Biomedical Optics*.

[8] Chinn SR, Swanson EA, Fujimoto JG (1997). Optical coherence tomography using a frequency-tunable optical source. *Optics Letters*.

[9] Brar M, Bartsch D-UG, Nigam N (2009). Colour versus grey-scale display of images on high- Resolution spectral OCT. *British Journal of Ophthalmology*.

[10] Han IC, Jaffe GJ (2010). Evaluation of artifacts associated with macular spectral-domain optical coherence tomography. *Ophthalmology*.

[11] Giani A, Cigada M, Esmaili DD (2010). Artifacts in automatic retinal segmentation using different optical coherence tomography instruments. *Retina*.

[12] Grover S, Murthy RK, Brar VS, Chalam KV (2009). Normative data for macular thickness by high-definition spectral-domain optical coherence tomography (spectralis). *American Journal of Ophthalmology*.

[13] Asefzadeh B, Cavallerano AA, Fisch BM (2007). Racial differences in macular thickness in healthy eyes. *Optometry and Vision Science*.

[14] Song WK, Lee SC, Lee ES, Kim CY, Kim SS (2010). Macular thickness variations with sex, age, and axial length in healthy subjects: a spectral domain-optical coherence tomography study. *Investigative Ophthalmology and Visual Science*.

[15] Pierro L, Giatsidis SM, Mantovani E, Gagliardi M (2010). Macular thickness interoperator and intraoperator reproducibility in healthy eyes using 7 optical coherence tomography instruments. *American Journal of Ophthalmology*.

[16] Patel N, Chowdhury H, Leung R, Sivaprasad S (2013). Sensitivity and specificity of time-domain versus spectral-domain optical coherence tomography in diabetic macular edema. *Indian Journal of Ophthalmology*.

[17] Schuman JS, Puliafito CA, Fujimoto JG (2004). *Optical Coherence Tomography of Ocular Diseases*.

[18] Wali UK, Al Kharousi N, Liu G (2012). Clinical applications of optical coherence tomography in ophthalmology. *Selected Topics in Optical Coherence Tomography*.

[19] Koleva-Georgieva D, Sivkova N (2009). Assessment of serous macular detachment in eyes with diabetic macular edema by use of spectral-domain optical coherence tomography. *Graefe’s Archive for Clinical and Experimental Ophthalmology*.

[20] Koleva-Georgieva DN, Sivkova NP (2008). Types of diabetic macular edema assessed by optical coherence tomography. *Folia Medica*.

[21] Otani T, Yamaguchi Y, Kishi S (2010). Correlation between visual acuity and foveal microstructural changes in diabetic macular edema. *Retina*.

[22] Maheshwary AS, Oster SF, Yuson RMS, Cheng L, Mojana F, Freeman WR (2010). The association between percent disruption of the photoreceptor inner segment-outer segment junction and visual acuity in diabetic macular edema. *American Journal of Ophthalmology*.

[23] Shin HJ, Lee SH, Chung H, Kim HC (2012). Association between photoreceptor integrity and visualoutcome in diabetic macular edema. *Graefe’s Archive for Clinical and Experimental Ophthalmology*.

[24] Yohannan J, Bittencourt M, Sepah YJ (2013). Association of retinal sensitivity to integrity of photoreceptor inner/outer segment junction in patients with diabetic macular edema. *Ophthalmology*.

[25] Patel JI, Hykin PG, Schadt M, Luong V, Fitzke F, Gregor ZJ (2006). Pars plana vitrectomy for diabetic macular oedema: OCT and functional correlations. *Eye*.

[26] Pendergast SD, Hassan TS, Williams GA (2000). Vitrectomy for diffuse diabetic macular edema associated with a taut premacular posterior hyaloid. *American Journal of Ophthalmology*.

[27] Shah SP, Patel M, Thomas D, Aldington S, Laidlaw DAH (2006). Factors predicting outcome of vitrectomy for diabetic macular oedema: results of a prospective study. *British Journal of Ophthalmology*.

[28] Gaucher D, Tadayoni R, Erginay A, Haouchine B, Gaudric A, Massin P (2005). Optical coherence tomography assessment of the vitreoretinal relationship in diabetic macular edema. *American Journal of Ophthalmology*.

[29] Otani T, Kishi S, Maruyama Y (1999). Patterns of diabetic macular edema with optical coherence tomography. *American Journal of Ophthalmology*.

[30] Kang SW, Park CY, Ham D-I (2004). The correlation between fluorescein angiographic and optical coherence tomographic features in clinically significant diabetic macular edema. *American Journal of Ophthalmology*.

[31] Panozzo G, Parolini B, Gusson E (2004). Diabetic macular edema: an OCT-based classification. *Seminars in Ophthalmology*.

[32] Kim BY, Smith SD, Kaiser PK (2006). Optical coherence tomographic patterns of diabetic macular edema. *American Journal of Ophthalmology*.

[33] Soliman W, Sander B, Jørgensen TM (2007). Enhanced optical coherence patterns of diabetic macular oedema and their correlation with the pathophysiology. *Acta Ophthalmologica Scandinavica*.

[34] Koleva-Georgieva D, Ola MS (2012). Optical coherence tomography findings in diabetic macular edema. *Diabetic Retinopathy*.

[35] Wang XJ, Milner TE, Nelson JS (1995). Characterization of fluid flow velocity by optical Doppler tomography. *Optics Letters*.

[36] Izatt JA, Kulkarni MD, Yazdanfar S, Barton JK, Welch AJ (1997). In vivo bidirectional color Doppler flow imaging of picoliter blood volumes using optical coherence tomography. *Optics Letters*.

[37] Zhao Y, Chen Z, Saxer C (2000). Doppler standard deviation imaging for clinical monitoring of in vivo human skin blood flow. *Optics Letters*.

[38] Wang Y, Fawzi AA, Varma R (2011). Pilot study of optical coherence tomography measurement of retinal blood flow in retinal and optic nerve diseases. *Investigative Ophthalmology and Visual Science*.

[39] Wang Y, Fawzi A, Tan O, Gil-Flamer J, Huang D (2009). Retinal blood flow detection in diabetic patients by Doppler Fourier domain optical coherence tomography. *Optics Express*.

